# A Ratiometric Fluorescence Probe for Visualized Detection of Heavy Metal Cadmium and Application in Water Samples and Living Cells

**DOI:** 10.3390/molecules29225331

**Published:** 2024-11-13

**Authors:** Qijiang Xu, Wen Qin, Yanfei Qin, Guiying Hu, Zhiyong Xing, Yatong Liu

**Affiliations:** 1 School of Laboratory Medicine, Youjiang Medical University for Nationalities, Baise 533000, China; qijiangxu@ymun.edu.cn (Q.X.); qinwen2023@126.com (W.Q.); 13557487915@139.com (Y.Q.); 13285362396@163.com (G.H.); 2 Modern Industrial College of Biomedicine and Great Health, Youjiang Medical University for Nationalities, Baise 533000, China; 3 Department of Chemistry, College of Arts and Sciences, Northeast Agricultural University, Harbin 150030, China

**Keywords:** fluorescence probe, ratiometric, heavy metal cadmium, smartphone-assisted, imaging living cells

## Abstract

Heavy metal cadmium (II) residuals have inflicted severe damage to human health and ecosystems. It has become imperative to devise straightforward and highly selective sensing methods for the detection of Cd^2+^. In this work, a ratiometric benzothiazole-based fluorescence probe (**BQFA**) was effortlessly synthesized and characterized using standard optical techniques for the visual detection of Cd^2+^ with a change in color from blue to green, exhibiting a significant Stokes shift. Moreover, the binding ratio of **BQFA** to Cd^2+^ was established as 1:1 by the Job’s plot and was further confirmed by FT-IR and ^1^HNMR titrations. The ratiometric fluorescence response via the ICT mechanism was confirmed by DFT calculations. Furthermore, the limit of detection for detecting Cd^2+^ was determined to be 68 nM. Furthermore, it is noteworthy that **BQFA** showed good performance in real water samples, paper strips, smartphone colorimetric identification, and cell imaging.

## 1. Introduction

Cadmium, a naturally occurring heavy metal found in the Earth’s crust, is known for its high toxicity, particularly in higher-valence chromium compounds, which may have carcinogenic effects and cause profound harm to living organisms and the environment [1,2,3]. Due to the widespread application of cadmium in the metallurgy, refractory, cast iron, and chemical industries and high-precision technology, it is released into the soil through industrial emissions and absorbed by plant roots, inhibiting the photosynthesis and growth of plants, with a metabolic half-life ranging from approximately 10 to 35 years [4,5,6,7,8,9]. Further aggregation in the human body through the food chain ultimately poses a threat to human health. In addition, the trace element cadmium may cause renal dysfunction, anemia, decreased immunity, skeletal softening, and neurological damage [10,11,12,13,14,15]. Meanwhile, excessive cadmium exposure contributes to a host of health issues, including kidney disease, lung disease, osteomalacia, and cardiovascular disorders [16,17,18,19]. Cadmium ions also disrupt the absorption and utilization of vital elements such as calcium and iron, thereby elevating the risk of lung cancer and other malignancies [20,21,22,23]. Of greater concern is the element’s non-biodegradability, allowing it to persist indefinitely in the ecosystem, with cadmium ions being notorious for generating free radicals in the human body and catalyzing the formation of lipid peroxides [24,25]. The USA Environmental Protection Agency classifies cadmium as the seventh most hazardous substance, and it was classified as a carcinogen to humans [26,27,28,29]. Consequently, the development and formulation of highly efficient and sensitive fluorescent probes for both qualitative and quantitative assessments of Cd^2+^ concentrations have emerged as a pressing necessity.

Numerous classical analytical methods have been applied to detect cadmium ions in environmental and biological samples, including electrochemical techniques, atomic absorption spectroscopy (AAS), inductively coupled plasma mass spectrometry (ICP-MS), high-performance liquid chromatography (HPLC), and tandem spectroscopic techniques (MS) [30,31,32,33,34]. However, these advanced technologies face certain limitations, such as high instrument costs, intricate sample preparation procedures, the requirement for skilled operators, limited selectivity during detection, and practical application challenges such as time consumption [35]. As a result, there is a growing demand for simple, rapid, and reliable techniques and materials for the on-site detection of Cd^2+^ ions. Fluorescent probes have shown great potential in monitoring heavy metal ions due to their high efficiency, ease of use, exceptional sensitivity, fast response times, and low detection limits. They have garnered significant attention in analytical chemistry, environmental science, and related fields [36,37,38,39,40]. Nevertheless, owing to the analogous physical and chemical properties shared by Cd^2+^ and other metal ions, eliminating interference during cadmium detection remains a considerable challenge for most currently available probes [41,42,43,44]. Developing Cd^2+^-selective fluorescent probes that can precisely identify Cd^2+^ among other closely related metal ions remains a significant challenge.

Moreover, recent studies in the literature have documented numerous fluorescent probes for Cd^2+^ ions [45,46,47,48]. However, the majority of these Cd^2+^ fluorescent probes have been investigated to primarily function through fluorescence enhancement [49,50], thus limiting their practical applications. Few ratiometric fluorescent molecular probes have been developed. Benzothiazole was chosen as the core structure for a probe’s design primarily because of its rigid, conjugated framework, which imparts excellent optical properties and a significant Stokes shift [51,52,53]. Moreover, nitrogen and oxygen atoms were incorporated into the structure of the probe as chelation sites to enhance its metal ion coordination capability. Ultimately, the C=N moiety of the Schiff base, a renowned ligand, was incorporated as well. In this case, the carbon–nitrogen double bond was further integrated into the target compound **BQFA**, resulting in significant variations in fluorescence signals during the detection of the specific analyte.

**Scheme 1:**
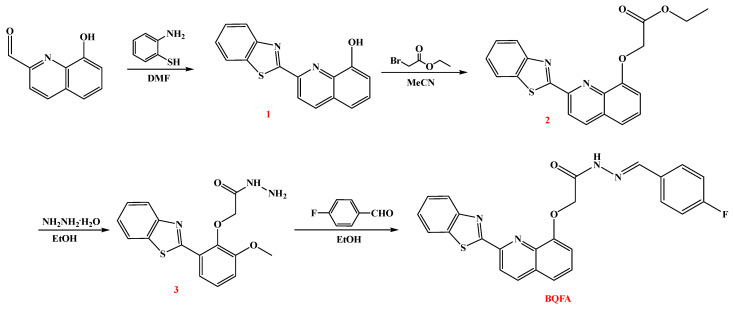
Synthetic route of **BQFA**.

The amalgamation of the aforementioned factors, as depicted in Scheme 1, resulted in a novel ratiometric fluorescence probe, (E)-2-((2-(benzo[d]thiazol-2-yl)quinolin-8-yl)oxy)-N′-(4-fluorobenzylidene) acetohydrazide (**BQFA**), which was derived from benzothiazole. It was effortlessly synthesized and meticulously characterized using conventional optical methods for the visual detection of Cd^2+^, demonstrating a pronounced color transition from blue to green, along with a notable Stokes shift. Additionally, the binding ratio of **BQFA** to Cd^2+^ was established as 1:1 by the Job’s plot and was further confirmed by Fourier transform infrared spectroscopy (FT-IR) and nuclear magnetic resonance hydrogen spectroscopy (^1^HNMR) titrations. The ratiometric fluorescence response via the intramolecular charge transfer (ICT) mechanism was confirmed by density functional theory (DFT) calculations. Furthermore, the detection limit (LOD) for Cd^2+^ was established at 68 nM. Notably, **BQFA** demonstrated excellent performance in real water samples, smartphone-assisted colorimetric identification, and A549 and Siha cell imaging.

## 2. Results and Discussion

### 2.1. Spectral Response of ***BQFA*** to Cd^2+^

**Figure 1:**
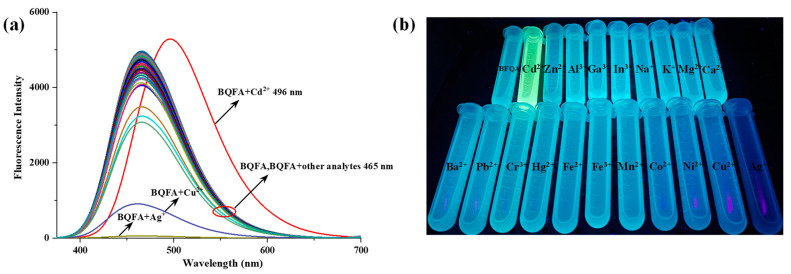
(**a**) Fluorescence spectra of **BQFA** (10 μM) in the presence of the tested analytes in DMF/H_2_O/EtOH (*v*/*v*/*v*, 1/1/98) solution (λ_ex_ = 350 nm). (**b**) Solution color change before and after the addition of Cd^2+^ (5 eq.).

In a mixed solution system, the selectivity of UV–Vis and fluorescence spectra and the corresponding solution color of **BQFA** (10 μM) were assessed with the addition of different analytes (56 analytes, including anions, ions, and amino acids). Firstly, the UV–Vis spectrum exhibited (Figure S1) that the absorption peaks of the probe **BQFA** were at 288 nm and 343 nm. Upon interaction with Cd^2+^ cations, significant spectral changes were observed; the UV absorption peak at 300 nm was weakened and blueshifted to 288 nm, while the UV absorption peak at 343 nm was enhanced and redshifted to 357 nm. The fluorescence spectra with the excitation and emission spectra of **BQFA** and **BQFA** + Cd^2+^ were measured (Figure S2), and the fluorescence spectra selectivity of **BQFA** was shown in Figure 1. The probe itself exhibits a Stokes shift of merely 115 nm at an excitation wavelength of 350 nm. Upon the incorporation of Cd^2+^, a significant redshift of the fluorescence emission peak was observed, increasing the Stokes shift to 146 nm, accompanied by a slight enhancement in fluorescence intensity (Figure 1a). Additionally, under ultraviolet illumination, as depicted in Figure 1b, the solution color transitioned from blue to bright green fluorescence. In contrast, the addition of other analytes resulted in neither a redshift nor an enhancement of the fluorescence emission spectrum nor any alteration in solution color under ultraviolet light. This indicated that probe **BQFA** demonstrates exceptional selectivity towards Cd^2+^.

The probe **BQFA** (10 μM) was tested in various organic solvents with different properties, and the fluorescence spectra of both the **BQFA** and the **BQFA** + Cd^2+^ complex were assessed. As shown in Figure S3, there were distinctions in the spectra of **BQFA** without (Figure S3a) and with Cd^2+^ (Figure S3b) in solvents of various polarities. The spectra showed a significant redshift accompanied by a change in the color of the solution in acetonitrile, methanol, ethanol, and acetone systems. Thus, these four solvents were the ones in which **BQFA** could specifically recognize Cd^2+^.

**Figure 2:**
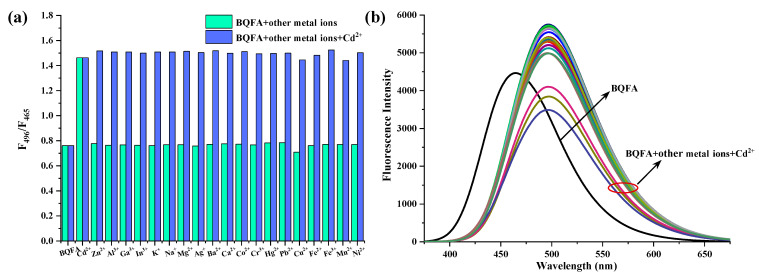
(**a**) Competition of fluorescence spectra of **BQFA** (10 μM) when detecting Cd^2+^ (5.0 eq.) in the presence of other metal ions (5.0 eq.) using the ratio of fluorescence intensity at 496 nm and 465 nm. (**b**) Fluorescence spectra of **BQFA** (10 μM) when detecting Cd^2+^ (5.0 eq.) in the presence of other metal ions (5.0 eq.).

In addition, competitive experiments were conducted to evaluate whether the probe **BQFA** could identify Cd^2+^ in a complex environment where multiple ions were present, as shown in Figure 2. Equal amounts of other metal ions were added to the solution of the probe **BQFA** (10 μM); then, the fluorescence spectra were measured, and the ratio of fluorescence intensity at 496 nm and 465 nm for Cd^2+^ was recorded, as shown in Figure 2a. As shown in Figure 2b, the addition of Cd^2+^ (50 μM) resulted in a significant redshift in the fluorescence intensity at 542 nm in the presence of other metal ions (50 μM). Therefore, none of the other metal ions could interfere with the recognition of Cd^2+^ by the probe **BQFA**, which indicated that the probe **BQFA** possessed excellent anti-interference ability when detecting Cd^2+^.

**Figure 3:**
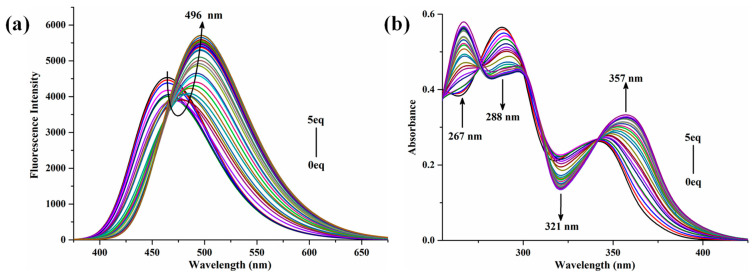
(**a**) Fluorescence titration of **BQFA** (10 μM) with the addition of Cd^2+^ (0–50 μM) in solution. (**b**) UV–Vis spectrum titration of **BQFA** (10 μM) with the addition of Cd^2+^ (0–50 μM) in solution.

To evaluate the sensitivity of the probe **BQFA** to Cd^2+^, UV–Vis and fluorescence spectra with concentration titration were further analyzed, as shown in Figure 3. The fluorescence titration experiment was performed at an excitation wavelength of 430 nm. As shown in Figure 3a, with increasing concentrations of Cd^2+^, the fluorescence emission peak of the probe **BQFA** solution progressively shifted from 465 nm to 496 nm, exhibiting a redshift. The fluorescence signal at 496 nm exhibited a linear relationship with the concentration of Cd^2+^ from 5 to 20 μM (R^2^ = 99,538) (Figure S4a), and the detection limit was calculated to be 3.2 × 10^−7^ M.

In the UV–Vis titration experiment, the UV absorption peaks of the probe **BQFA** solution at 267 nm increased with the increase in Cd^2+^ concentration (Figure 3b), while the absorption peak at 343 nm gradually decreased and redshifted to 357 nm. Remarkable isobestic points were found at 276 nm and 343 nm. Additionally, it can be seen that the absorbance ratio (A_357_/A_321_) was related to Cd^2+^ (0–20 μM) in Figure S4b. The concentration had a good linear relationship (R^2^ = 0.99538), and the limit of detection was calculated to be 6.80 × 10^−8^ M according to the 3σ/S value. These results indicated that the probe **BQFA** is capable of quantitatively detecting Cd^2+^.

### 2.2. Mechanism Research of ***BQFA*** with Cd^2+^

**Figure 4:**
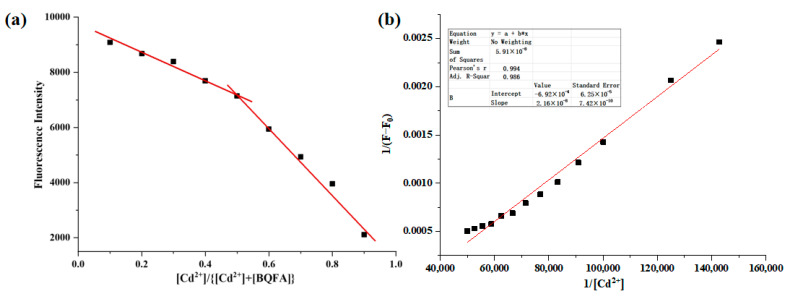
(**a**) Job’s plot of **BQFA** and Cd^2+^. (**b**) Benesi–Hildebrand plot of **BQFA** with Cd^2+^.

The binding stoichiometry of the probe **BQFA** toward Cd^2+^ was assessed using Job’s plot for fluorescent titration (Figure 4). The total concentration of the probe **BQFA** and Cd^2+^ was kept constant at 1 × 10^−5^ M, and the test solutions were prepared in accordance with different ratios of [Cd^2+^]/{[**BQFA**] + [Cd^2+^]}. The fluorescence intensity at 496 nm was measured, as indicated in Figure 4a, and the results indicated that the complexation ratio of the probe **BQFA** to Cd^2+^ was 1:1. Further analysis involved plotting the Benesi–Hildebrand equation curve (Figure 4b), from which the complexation constants (K) between the probe **BQFA** and Cd^2+^ were calculated. The association constant was determined to be 5.21 × 10^4^ M^−1^.

Subsequently, the complexation mode of **BQFA** with Cd^2+^ was further demonstrated by assessing the HRMS spectrum. As depicted in Figure S5, the peak at 631.9967 was designated as [**BQFA** + Cd^2+^ + NO_3_^−^]^+^ (theoretical value: 631.9968). These results reinforced the evidence that the complexation ratio between the probe **BQFA** and Cd^2+^ was 1:1.

The FT-IR spectra of **BQFA** both prior to and following the addition of Cd^2+^ were analyzed to elucidate the intricacies of the coordination between **BQFA** and Cd^2+^ (Figure S6). The peaks at 3315, 1705, and 1334 cm^−1^ were ascribed to the stretching vibrations of -NH, C=O, and C=N. The peaks of -NH, C=O, and C=N redshifted to 3257, 1687, and 1303 cm^−1^ in the FT-IR spectrum of **BQFA**-Cd^2+^.

Moreover, ^1^H NMR titrations were conducted to elucidate the intricate details of the binding of **BQFA** to Cd^2+^ (Figure S7) in the solution of DMSO-d_6_. The integral region of H_a′_ and H_a_ in the **BQFA** of the ^1^H NMR titration spectra, as depicted in Figure S8, exhibited a subtle increase in the ratio of peak areas corresponding to the two isomers with the incremental addition of Cd^2+^. The protons of the amide group (designated as H_a_ and H_a′_) and the protons (designated at H_b_ and H_b′_) of the carbon–nitrogen double bond correspond to (*Z*)- and (*E*)-configurations arising from the C=N isomerization of the probe **BQFA** [54]. The proton signal (H_a_) at 11.71 ppm was broadened, and there was a more obvious split with the addition of Cd^2+^. Further, the proton signals (H_b_) and (H_c_) were broadened and moved toward the high field. All of this indicated that the oxygen atom on the amide and the nitrogen atom on the carbon–nitrogen double bond of the probe **BQFA** combined with Cd^2+^ and composed the complex.

**Figure 5:**
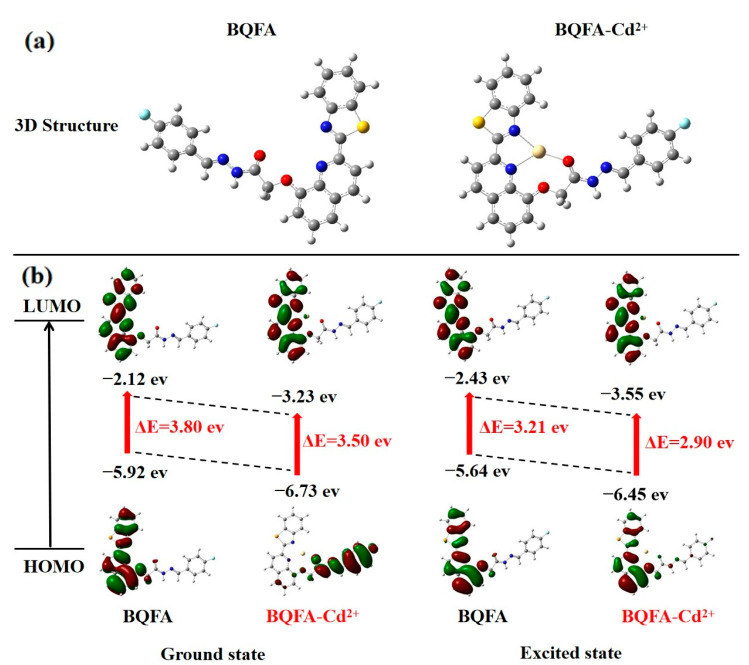
(**a**) Energy-optimized structure of the probe **BQFA** and **BQFA**+Cd^2+^. (**b**) Calculated molecular orbitals and the HOMO–LUMO gaps of **BQFA** and **BQFA** + Cd^2+^ in the ground state and excited state.

Quantum chemical calculations of the binding model for the **BQFA** and **BQFA**-Cd^2+^ complex on the theoretical level were conducted using density functional theory (DFT) to confirm the combination mechanism (Figure 5). The optimized three-dimensional structure of **BQFA** and the **BQFA**-Cd^2+^ complex are illustrated in Figure 5a. Correspondingly, the energy optimization of the highest occupied molecular orbital (HOMO) and the lowest unoccupied molecular orbital (LUMO) for **BQFA** and the **BQFA**-Cd^2+^ complex in both the ground and excited states was revealed (Figure 5b). The energy gaps between the HOMO and LUMO of **BQFA** and **BQFA**-Cd^2+^ were determined to possess energy levels of 3.80 eV and 3.50 eV in their ground states and 3.21 eV and 2.89 eV in their excited states, respectively. These discoveries inclined toward the proposition that the intermingling between the probe **BQFA** and Cd^2+^ significantly reduced the HOMO–LUMO energy gap of **BQFA** and stabilized the sensory system, further correlating well with the pronounced redshift observed in the UV–Vis spectra of the **BQFA**-Cd^2+^ complex compared with free **BQFA** [55,56].

**Scheme 2:**
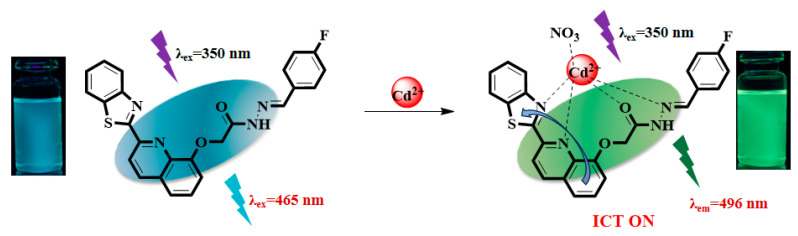
Probable interaction mechanism of **BQFA** and Cd^2+^.

According to the above experimental results obtained using Job’s plot, HRMS FT-IR titration, ^1^HNMR titration, and the DFT study, the probable mechanism of the interaction of **BQFA** with Cd^2+^ was elucidated based on the ICT mechanism, as shown in Scheme 2. Upon the coordination of **BQFA** with Cd^2+^, the ICT mechanism was triggered, causing a redshift in the fluorescence emission spectrum and a notable transition from blue to green in the solution.

### 2.3. Reversibility Studies

The reversibility of fluorescent probes is crucial for evaluating their efficacy in practical applications. Accordingly, reversibility assessments of the UV–Vis and fluorescent spectra were measured by alternately adding the Cd^2+^ (10 μM) and EDTA (10 μM) to the **BQFA** solution, which was designated as one cycle, and the results were illustrated in Figure S9a,b. For the first cycle, the introduction of Cd^2+^ (10 μM) into the probe **BQFA** (10 μM) solution resulted in a redshift in both the UV–Vis and fluorescent spectra. After adding EDTA (10 μM) to the solution, the UV–Vis absorption spectra and fluorescence spectra of the solution of **BQFA** were all nearly recovered to the original state of **BQFA** in the absence of Cd^2+^, with the solution color changed from green to blue. However, for the second cycle, after the additions of Cd^2+^, the tendency of the redshift of the spectra (including the UV–Vis and fluorescent spectra) all decreased, and the spectrum reverted to its original state after the addition of EDTA to the mixture. As for the third cycle, the additions of Cd^2+^ caused a slight redshift change in the UV–Vis absorption spectra and fluorescence spectra of **BQFA** and reverted to the original state after the addition of EDTA. These results showed that the reversibility of **BQFA** was limited within three cycles and that it could also be used in the manufacture of devices for the detection of Cd^2+^.

## 3. Practical Applications

### 3.1. Application on Test Paper and in a Smartphone

**Figure 6:**
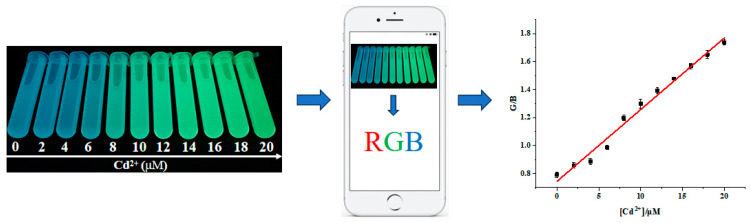
Schematic representation of an RGB analysis of images produced via a color recognition application.

The color contrasts of **BQFA** solutions under UV light, upon the introduction of varying quantities of Cd^2+^ (0–20 μM), were distinctly evident, transitioning from blue to cyan, then to green, and ultimately to yellow-green. This transformation could be discerned using a smartphone application known as “Color Recognizer” for colorimetry. As shown in Figure 6, the RGB values of the color **BQFA** correspond to sample solutions with fluorescence colorimetry under UV light. With the increase in the addition of Cd^2+^ (0–20 μM), the G/B ratio of the solutions was found to have a good linear relationship, the R^2^ value was 0.98353, and the LOD was determined to be 1.12 μM. The findings reveal that, with the assistance of a smartphone, probe **BQFA** is capable of accurately quantifying the concentration of Cd^2+^ through the RGB values corresponding to the color of the solution. The test paper was immersed in **BQFA** solution for 2 h and then dried. The fluorescent color changed from colorless to bright green upon the addition of Cd^2+^ to the paper (Figure S10), indicating that **BQFA** has the potential for portable Cd^2+^ detection using the test paper method.

### 3.2. Analysis of Cd^2+^ in Real Samples

To demonstrate the practical use of the probe **BQFA** in metal ions, experiments on the Cd^2+^ levels in tap water, water from the You River, and an aqueous solution of soil were conducted [57]. The emission intensity of **BQFA** at 496 nm for the three aqueous samples was separately transcribed after adding different concentrations of Cd^2+^ (5 μM, 10 μM, and 15 μM). The relevant data were then calculated and tabulated in Table 1 (*n* = 3). The Cd^2+^ recovery rate in the three water samples was satisfactory, ranging from 97.5% to 103.1%. In conclusion, the fluorescent probe **BQFA** demonstrated its feasibility and potential for detecting Cd^2+^ with high accuracy in actual water samples. Since the LOD of the probe, **BQFA** was currently unable to reach the concentration of Cd^2+^ allowed by the WHO in tap water, and the **BQFA** was more suitable for measuring the content of Cd^2+^ in water bodies that were heavily polluted with heavy metal cadmium.

### 3.3. Fluorescence Imaging of Cd^2+^ in Living Cells

**Figure 7:**
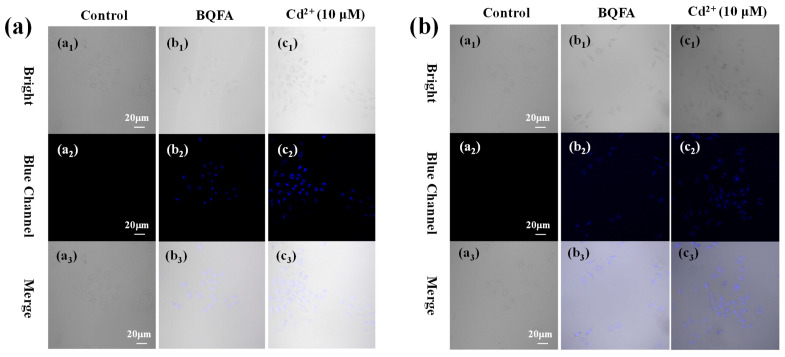
(**a**) Fluorescence microscopy images of A549 cells interacting with **BQFA** in the presence of Cd^2+^: (a_1_–a_3_) Cells imaged after treatment with PBS buffer solution; (b_1_–b_3_) cells after 1 h of incubation with 10 µM **BQFA**; (c_1_–c_3_) cells exposed to 10 µM Cd^2+^ for 1 h, followed by 1 h of incubation with 10 µM **BQFA** for imaging. (**b**) Fluorescence microscopy images of Siha cells interacting with **BQFA** in the absence and presence of Cd^2+^: (a_1_–a_3_, b_1_–b_3_, and c_1_–c_3_) imaging was conducted in the blue channel, with an excitation wavelength (λ_ex_) of 405 nm and an emission range (λ_em_) of 500–550 nm. Scale bar: 20 μm.

These experiments were motivated by the exceptional sensing capabilities of the probe **BQFA** for Cd^2+^ within biosystems. The experimental outcomes are presented in Figure 7, demonstrating that when using the probe **BQFA** to detect Cd^2+^ in living A549 and Siha cells, there was a specific change in the fluorescent signal within the cytoplasm. The probe **BQFA** (10 μM) itself showed weak fluorescence (Figure 7a(b_2_),b(b_2_)) compared with the outcomes of the comparative experiment (Figure 7a(c_2_),b(c_2_)), which demonstrated a marked enhancement in fluorescence following the cultivation of the cells with **BQFA** and Cd^2+^ (10 μM). This observation implied that **BQFA** possessed the ability to specifically target Cd^2+^ in these biological investigations.

## 4. Materials and Methods

### 4.1. Experiments

#### 4.1.1. Synthesis of **BQFA**

The synthesis of the target compound **BQFA** is displayed in Scheme 1. The synthesis methodology for compounds **1**–**3** was carried out with reference to a previously reported procedure [58]. Compound **3** (141.0 mg, 0.4 mmol) and 4-fluorobenzaldehyde (49.6 mg, 0.4 mmol) were dissolved in 20 mL ethanol, followed by the addition of a catalytic amount of acetic acid. Then, they were stirred and refluxed until TLC monitoring indicated the reaction’s completion. The reaction was stopped, the reaction liquid was chilled to room temperature, and the precipitate was isolated through filtration and subsequently washed multiple times with ethanol to procure the pure target compound, which was **BQFA** as a white powder. Yield: 72.8%. m.p. 240.7–241.1 °C. IR (KBr, *ν_max_*, cm^−1^) (Figure S11): 3315 (N-H), 1705 (C=O). ^1^H NMR (600 MHz, DMSO-*d_6_*) (Figure S12) *δ* (ppm) 11.71 (s, 1H), 8.57 (d, J = 8.6 Hz, 1H), 8.47 (d, J = 8.6 Hz, 1H), 8.18 (d, J = 7.3 Hz, 1H), 8.16 (d, J = 3.6 Hz, 1H), 8.05 (s, 1H), 7.82 (dd, J = 8.4, 5.6 Hz, 1H), 7.77 (dd, J = 8.4, 5.6 Hz, 1H), 7.65 (d, J = 8.8 Hz, 1H), 7.60 (t, J = 7.7 Hz, 2H), 7.53 (t, J = 7.5 Hz, 1H), 7.34–7.30 (m, 1H), 7.28 (t, J = 8.5 Hz, 1H), 7.23 (d, J = 7.7 Hz, 1H), 5.57 (s, 1H), 5.04 (s, 1H). ^13^C NMR (151 MHz, DMSO-*d_6_*) (Figure S13) δ (ppm) 169.9, 169.4, 154.4, 154.3, 149.6, 143.2, 139.7, 138.2, 136.3, 131.1, 131.1, 130.5, 129.7, 129.6, 128.8, 127.2, 126.7, 124.0, 123.1, 120.6, 118.7, 116.4, 116.3, 112.3, 66.5. HRMS (Figure S14): *m*/*z* (TOF MS ES^+^): Calcd. for [C_25_H_18_FN_4_O_2_S]^+^: 457.1134 [**BQAF** + H^+^]^+^, found: 457.1130.

#### 4.1.2. Spectral Measurement Preparation

The stock solutions of various metal ions (10 mM) (Metal ions: Hg^2+^, K^+^, Na^+^, Zn^2+^, Ca^2+^, Mg^2+^, Al^3+^, Fe^2+^, In^3+^, Ba^2+^, Co^2+^, Cr^3+^, Pb^2+^, Cu^2+^, Fe^3+^, Mn^2+^, Ni^2+^, Ga^3+^, and Cd^2+^. Anion ions: F^−^, Cl^−^, Br^−^, I^−^, S^2−^, H_2_PO_4_^−^, HCO_3_^–^, OH^–^, SCN^-^, CH_3_COO^–^, ClO_4_^−^, BF_4_^−^, HPO_4_^2−^, CO_3_^2–^, SO_3_^2−^, HSO_3_^−^, SO_4_^2−^, HSO_4_^−^, S_2_O_3_^2−^, NO_2_^–^ and NO_3_^–^. Amino acids: Met, Ser, Arg, Lys, Ala, Asn, Val, Asp, Glu, Gly, His, Phe, Gln, GSH, and Cys) were prepared by dissolving accurately weighed components in ultrapure water. The test solution of probe **BQFA** (1 mM) was diluted in dimethylformamide (DMF) and further diluted into a mixed solvent of DMF/H_2_O/EtOH (*v*/*v*/*v*, 1/1/98) to achieve a final concentration of 10 μM, necessary for the acquisition of UV–Vis and fluorescence spectra. The excitation wavelength for the fluorescence spectra was set at 350 nm, with both the excitation and emission slit widths adjusted to 10 nm.

#### 4.1.3. Spectral Measurement Preparation and Applications of **BQFA**

The details of the materials, apparatuses, instruments, experimental conditions, and methods for the practical applications are delineated in the Supplementary Materials of this study.

## 5. Conclusions

We successfully synthesized a benzothiazole-based ratiometric fluorescence probe, **BQFA**, for the visual detection of Cd^2+^, exhibiting a pronounced color variation from blue to green with a notable Stokes shift. Job’s plot, FT-IR and ^1^H NMR titration, and DFT calculations were examined to verify the 1:1 ratio of the combination between **BQFA** and Cd^2+^ and the mechanism of action of ICT. With a detection limit of 68 nM, **BQFA** proved highly effective in detecting Cd^2+^ in various applications, including real water samples, paper strips, smartphone colorimetric identification, and cell imaging, highlighting its potential as a practical tool for environmental and biological monitoring.

## Figures and Tables

**Table 1:** Determination of Cd^2+^ in water samples from different water sources.

Water Samples Studied	Amount of Standard Cd^2+^ Added (μM)	Total Cd^2+^ Found (*n* = 3) (μM)	Recovery of Cd^2+^ (*n* = 3) Added (%)	Relative Error (%)
Tap water	5	4.97 ± 0.23	99.4	4.62
	10	10.21 ± 0.19	102.1	1.86
	15	14.87 ± 0.21	99.1	1.41
You River water	5	5.08 ± 0.15	101.6	2.95
	10	10.13 ± 0.31	103.1	3.06
	15	14.96 ± 0.33	99.7	2.20
Aqueous solution of soil extract	5	5.22 ± 0.37	104.4	7.08
	10	9.75 ± 0.28	97.5	2.87
	15	15.31 ± 0.45	102.1	2.94

## Data Availability

Data will be provided upon request to the corresponding author.

## Supplementary Materials

The following supporting information can be downloaded at https://www.mdpi.com/article/10.3390/molecules29225331/s1. Figure S1. Absorption spectra of **BQFA** (10 μM) without and with Cd^2+^ (5.0 eq.) in solution. Figure S2. (a) The excitation (Em = 465 nm) and emission (Ex = 350 nm) fluorescence spectra of **BQFA**; (b) The excitation (Em = 496 nm) and emission (Ex = 350 nm) fluorescence spectra of **BQFA** + Cd^2+^. Slit width: Ex = 2.0 nm; Em = 2.0 nm. Figure S3. (a) Fluorescence spectra of BQFA (10 μM) in different solvents. Inset: solution color change of **BQFA** (10 μM) in different solvents. (b) Fluorescence spectra of **BQFA** (10 μM) in different solvents. Inset: solution color change of **BQFA** (10 μM) with the addition of Cd^2+^ (5 eq.) in different solvents. Inset: solution color change of **BQFA** (10 μM) with the addition of Cd^2+^ (5 eq.) in different solvents. Figure S4. (a) Linearity of fluorescence intensity of **BQFA** (10 μM) with addition of Cd^2+^ (5–20 μM) at 496 nm. (b) Linearity of the ratio of absorption of **BQFA** (10 μM) with addition of Cd^2+^ (0–20 μM) at 357 nm and 321 nm. Figure S5. HRMS spectrum of **BQFA** upon addition of Cd^2+^. Figure S6. FT-IR spectra of **BQFA** and the **BQFA**-Cd^2+^ complex. Figure S7. ^1^H NMR titration spectra of **BQFA** with different concentrations of Cd^2+^. Figure S8. The integral area of Ha’ and Ha in **BQFA** of ^1^H NMR titration spectra with different concentrations of Cd^2+^. Figure S9. (a) The fluorescence spectra of **BQFA** (10 μM) upon the alternate addition of Cd^2+^ and EDTA. Inset: The color changes in **BQFA** (10 μM) upon the alternate addition of Cd^2+^ and EDTA under a UV lamp at 365 nm. (b) The UV-Vis spectra of **BQFA** upon the alternate addition of Cd^2+^ and EDTA. Figure S10. Fluorescence color change after dropping Cd^2+^ onto the test paper treated with **BQFA**. Figure S11. FT-IR of compound **BQFA**. Figure S12. ^1^H NMR of compound **BQFA**. Figure S13. ^13^C NMR of compound **BQFA**. Figure S14. HRMS of probe **BQFA**.
