# Supplementary material for: A Ratiometric Fluorescence Probe for Visualized Detection of Heavy Metal Cadmium and Application in Water Samples and Living Cells
Source: Molecules. 2024 Nov 13;29(22):5331. doi: 10.3390/molecules29225331 (PMC11596035; doi:10.3390/molecules29225331)
Supplement: Supplementary file 1 [file molecules-29-05331-s001.zip › molecules-3250426-supplementary.pdf]

Supporting Information

A Ratiometric Fluorescence Probe for Visualized Detection of Heavy Metal Cadmium and Application in Water Samples and Living Cells

Qijiang Xu [1,2,†], Wen Qin [1,†], Yanfei Qin [1,2], Guiying Hu [1], Yatong Liu [3,*] and Zhiyong Xing [1,2,*]

[1] School of Laboratory Medicine, Youjiang Medical University for Nationalities, Baise 533000, China; qijiangxu@ymun.edu.cn (Q.X.); qinwen2023@126.com (W.Q.); 13557487915@139.com (Y.Q.); 13285362396@163.com (G.H.)
[2] Modern Industrial College of Biomedicine and Great Health, Youjiang Medical University for Nationalities, Baise 533000, China
[3] Department of Chemistry, College of Arts and Sciences, Northeast Agricultural University, Harbin 150030, China
* Correspondence: liuyatong@neau.edu.cn (Y.L.); zyxing@ymun.edu.cn (Z.X.)
† These authors contributed equally to this work.

Contents

1. Materials and methods 3

2. DFT Theoretical calculations of BQFA 3

3. Paper strips of BQFA 4

4. Detection of Smartphone platform for BQFA 4

5. Detection of Cd^{2+} in real water samples 4

6. Detection of Cd^{2+} in living cells 5

Figure. S1 5

Figure. S2 6

Figure. S3 6

Figure. S4 7

Figure. S5 7

Figure. S6 8

Figure. S7 8

Figure. S8 9

Figure. S9 9

Figure. S10 10

Figure. S11 10

Figure. S12 11

Figure. S13 11

Figure. S14 12

References 12

1. Materials and instruments

All chemical reagents were purchased with analytical or spectroscopic grade from commercial source of Aladdin Bio-Chem Technology Co., Ltd. and used as received. Bruck AV-600 spectrometer (Bruker, Germany) was used to record ^{1}H NMR and ^{13}C NMR spectra with TMS and DMSO-d_6 as the solvent. The chemical structural information of the compound was measured on ESI-MS and FT-IR spectra obtained on Waters Xevo UPLC/Xevo G2-XS Q-Tof MS spectrometer (Waters, America) and Bruker SEWERE ALPHA-T (Bruker, Germany), respectively. The absorption and fluorescent spectra data were collected by Shimadzu UV-2550 UV–vis spectrometer (Shimadzu, Japan) and Hitachi F-4700 fluorescence spectrometer (Hitachi, Japan) at 25 °C. Photographs were taken under a Qinke ZF-1A UV analyzer (Shanghai, China) using a smartphone (Huawei, China).

2. DFT Theoretical calculations of BQFA

Theoretical calculations were conducted using the modern density functional theory (DFT) method and the B3LYP/6-31G method within the Gauss Version 5.0.8 program [1]. The structure was optimized without any symmetric constraints, the DFT method was used to optimize the ground state geometry of **BQFA** and its complexes with Cd^{2+} (**BQFA**-Cd^{2+}). The leading molecular orbitals (FMOs) and HOMO-LUMO energy gap plots of **BQFA** and **BQFA**-Cd^{2+} the respective molecular systems were also calculated at the Gaussian theoretical level. The Gauss View package was utilized to generate color-filled iso-surface graphs for visualizing molecular orbitals [2]. And the energy calculations for all elements were obtained with the 6-31G (d, p) base set.

3. Paper strips of BQFA

The discs of the same size test paper were soaked in probe **BQFA** (10 μM) solution for 30 min and removed to air dry. Then these paper strips were treated with Cd^{2+} prepared in pure aqueous media. The differences were observed under a 365 nm UV lamp.

4. Detection of Smartphone platform for BQFA

The characteristic photograph of **BQFA** solutions (10 μM) with varying concentrations of Cd^{2+} under 365 nm UV lamp were captured using a smartphone. The green and blue channel values were directly obtained via an application on the smartphone, which could discern the G/B ratio for each Cd^{2+} concentration in the samples through fluorescence colorimetry. Consequently, visualization and quantitative analysis of Cd^{2+} could be achieved with the aid of a smartphone.

5. Detection of Cd^{2+} in real water samples

The detection of Cd^{2+} was conducted on three real samples, tap water, You River water, and soil extraction solution. For this, the sensor **BQFA** stock solution was mixed with the respective test sample in DMF/H_2O/EtOH (v/v/v, 1/50/49). The different concentrations of Cd^{2+} (5, 10, 15 μM) were added to prepare spiked samples for the test. Finally, recovery rates and relative errors were calculated.

In a 50 mL centrifuge tube containing soil sample weighing 5.0 g, acetonitrile 5 mL, sodium hydrogen sulfate 0.25 g, and water 2.5 mL were added, where the mixture was then sonicated for 5 min. Subsequently, sodium chloride (0.3 g) and anhydrous magnesium sulfate (0.5 g) were introduced, followed by thorough homogenization of the solid-liquid mixture. After centrifuging filtrate (8000 r/min) for 5 min, supernatant (5 mL) was extracted for subsequent purification. Afterward, 50 mg of anhydrous magnesium sulfate and 25 mg of PSA were used to clean the supernatant, followed by another round of mixing for 1 min. Soil sample underwent another round of centrifugation at 8000 r/min for 5 min. Ultimately, 5 mL soil extraction solution were filtered using a 0.45 μM filter for analysis. The resultant filtrate was diluted to 100 mL to create a soil extract solution with a concentration of 0.01 g/mL, primed for further experiments or analysis [3].

6. *Detection of Cd^{2+} in living cells*

A549 and Siha cells were obtained from iCell Bioscience Inc, Shanghai. The cells were divided into four groups as follows, control, probe **BQFA**, probe **BQFA**+Cd^{2+}. All groups were added 1 mL of PBS to wash twice, then added 1 mL of serum-free medium, incubated for 1 h. After that, the medium was sucked off, and 1 mL serum-free medium containing 20 and 50 μM Cd^{2+} ions was added according to the experimental group, respectively. After incubation for 1 h, the culture solution was removed, the cells were washed with PBS for three times, then added and cultured for 1 h with 4% paraformaldehyde (1 mL/dish), which was freshly prepared and fixed at room temperature. After 3 times washing with PBS, 1 mL probe solution (10 μM) was added and the cells were incubated for 2 hours, then washed with PBS for 3 times.

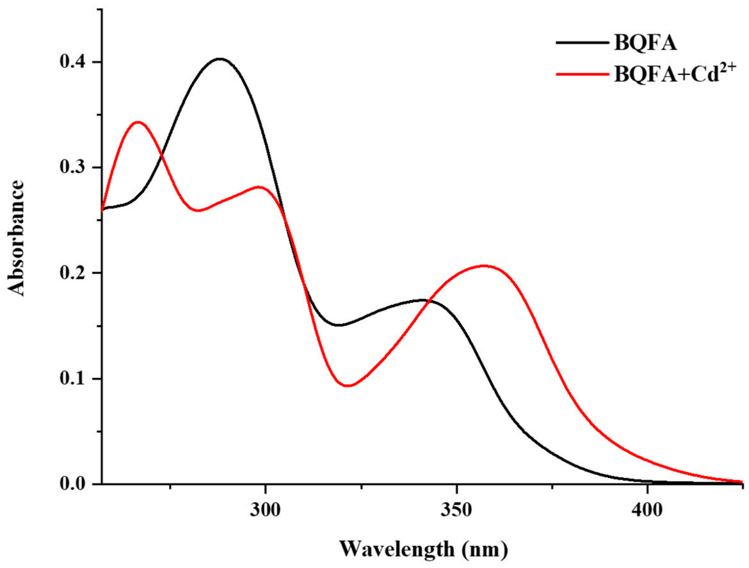

Figure. S1. Absorption spectra of **BQFA** (10 μM) without and with Cd^{2+} (5.0 eq.) in solution.

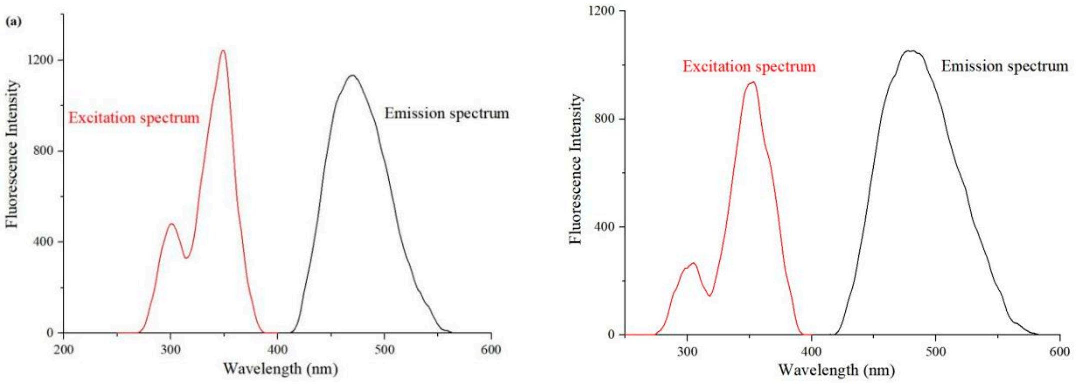

Figure. S2. (a) The excitation (E_m=465 nm) and emission (E_x=350 nm) fluorescence spectra of **BQFA;** (b) The excitation (E_m=496 nm) and emission (E_x=350 nm) fluorescence spectra of **BQFA**+Cd^{2+}. Slit width: E_x=2.0 nm; E_m=2.0 nm.

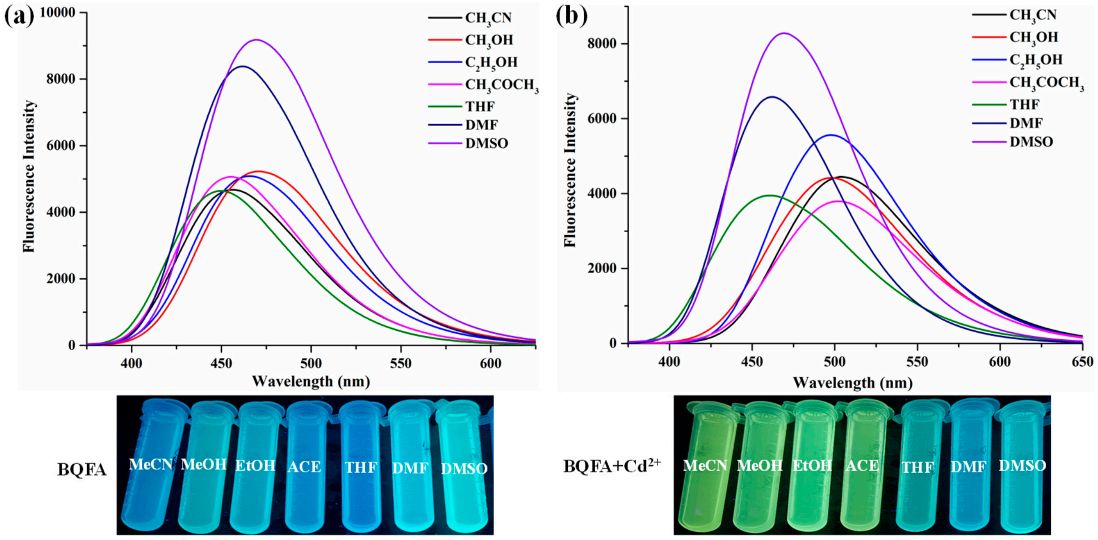

Figure. S3. (a) Fluorescence spectra of **BQFA** (10 μM) in different solvents. Inset: solution color change of **BQFA** (10 μM) in different solvents. (b) Fluorescence spectra of **BQFA** (10 μM) in different solvents. Inset: solution color change of **BQFA** (10 μM) with the addition of Cd^{2+} (5 eq.) in different solvents. Inset: solution color change of **BQFA** (10 μM) with the addition of Cd^{2+} (5 eq.) in different solvents.

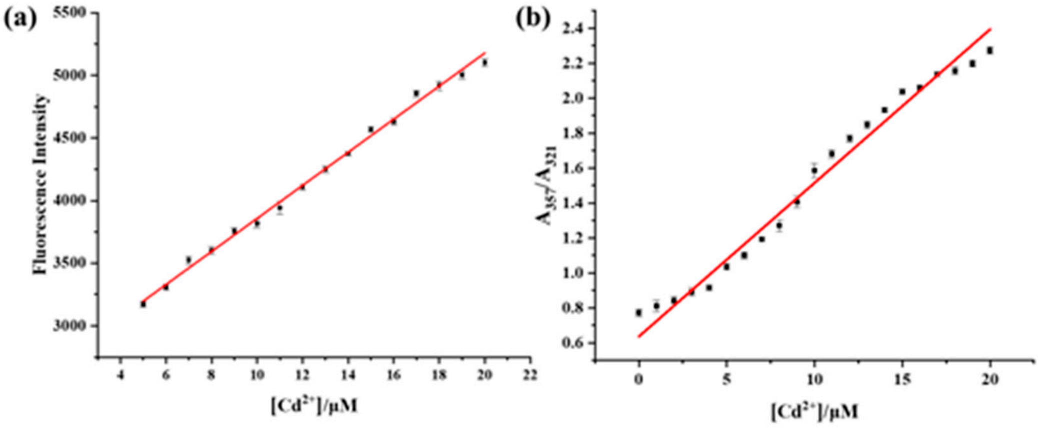

Figure. S4. (a) Linearity of fluorescence intensity of **BQFA** (10 μM) with addition of Cd^{2+} (5-20 μM) at 496 nm. (b) Linearity of the ratio of absorption of **BQFA** (10 μM) with addition of Cd^{2+} (0-20 μM) at 357 nm and 321 nm.

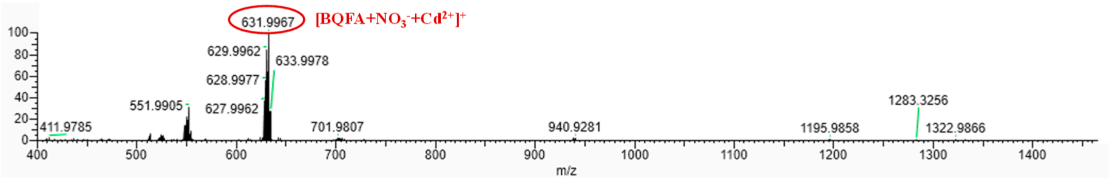

Figure. S5. HRMS spectrum of **BQFA** upon addition of Cd^{2+}.

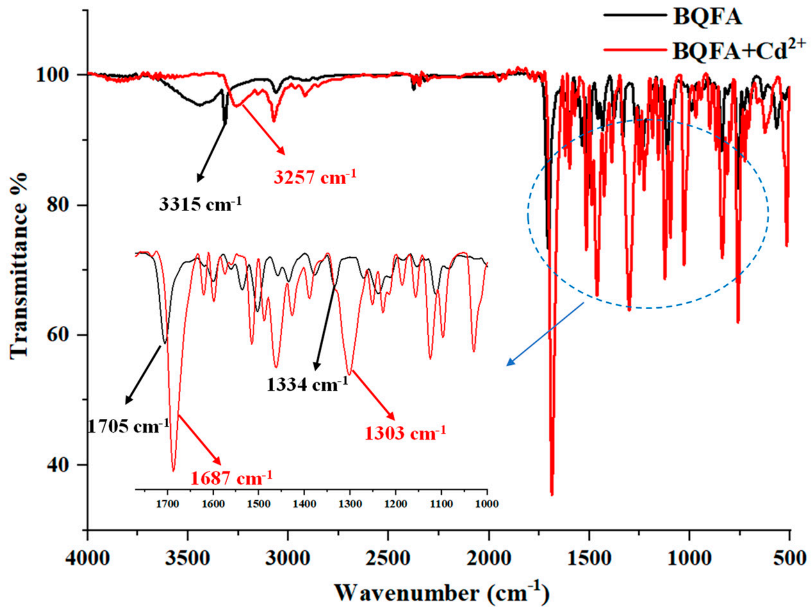

Figure. S6. FT-IR spectra of **BQFA** and the **BQFA**-Cd^{2+} complex.

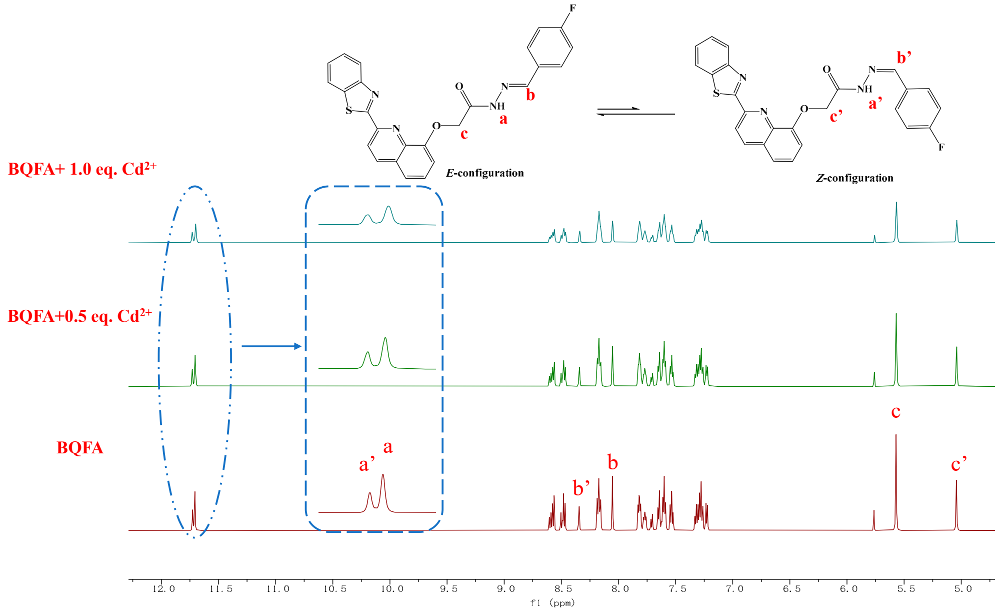

Figure. S7. ^{1}H NMR titration spectra of **BQFA** with different concentrations of Cd^{2+}.

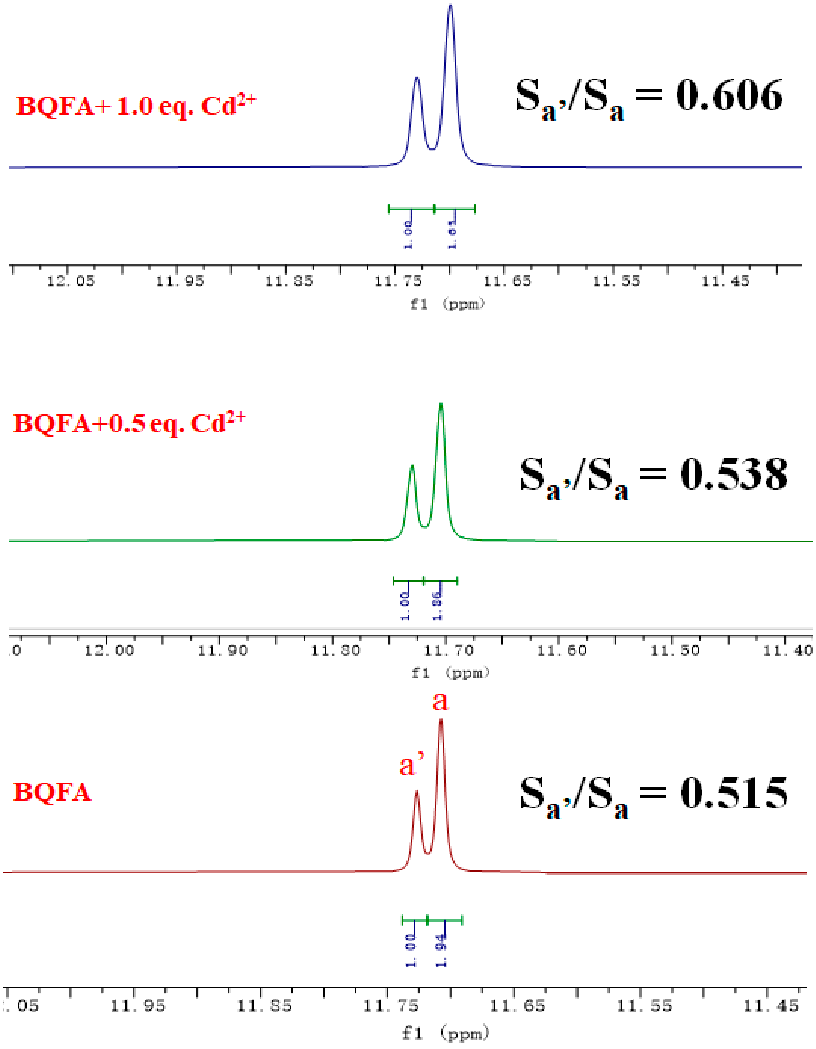

Figure. S8. The integral area of $H_{a'}$ and H_a in **BQFA** of ^1H NMR titration spectra with different concentrations of Cd^{2+}.

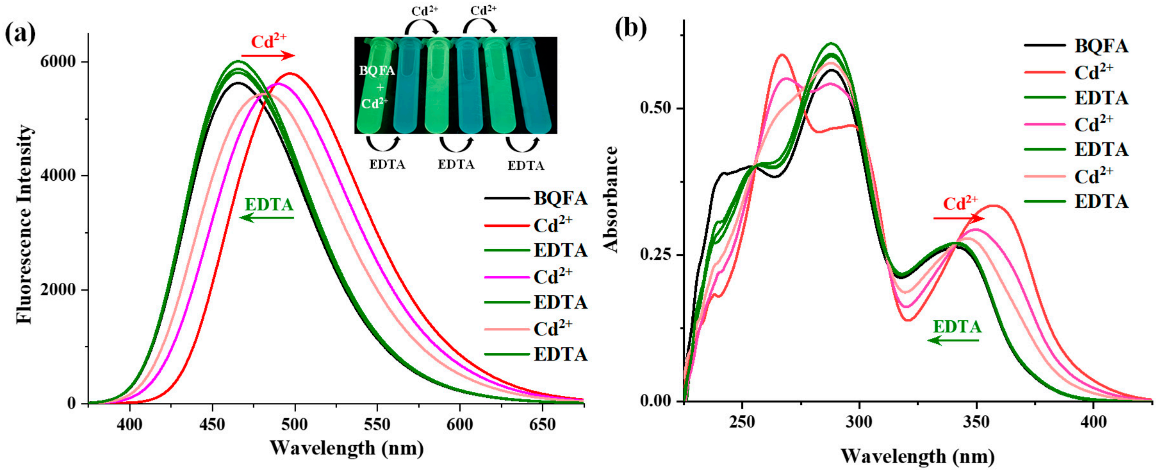

Figure S9. (a) The fluorescence spectra of **BQFA** (10 μM) upon the alternate addition of Cd^{2+} and

EDTA. Inset: The color changes in **BQFA** (10 μM) upon the alternate addition of Cd^{2+} and EDTA under a UV lamp at 365 nm. (b) The UV-Vis spectra of **BQFA** upon the alternate addition of Cd^{2+} and EDTA.

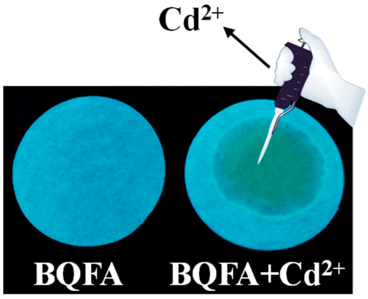

Figure. S10. Fluorescence color change after dropping Cd^{2+} onto the test paper treated with **BQFA**.

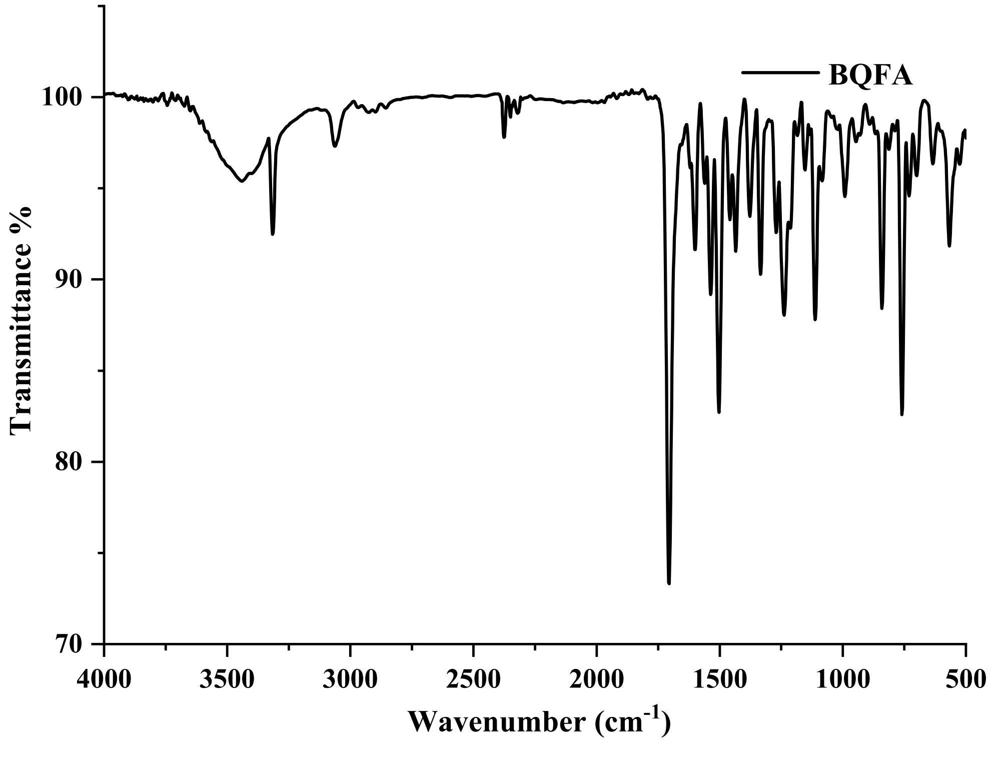

Figure. S11. FT-IR of compound **BQFA**.

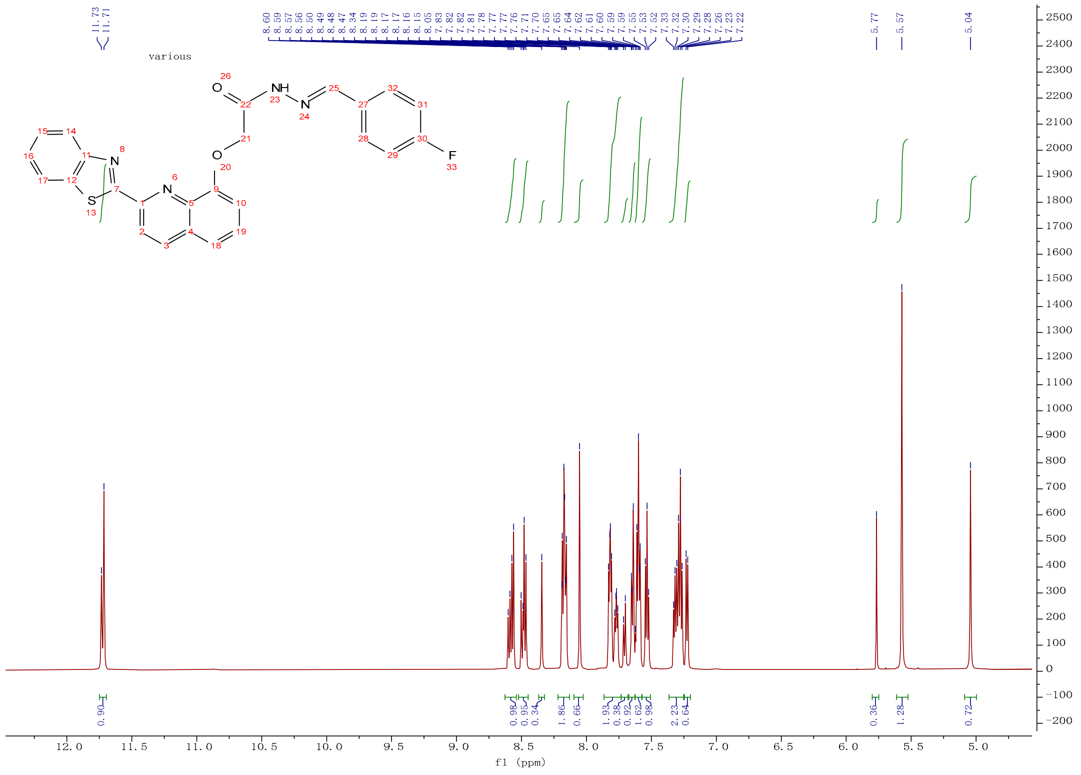

Figure. S12. ^{1}H NMR of compound **BQFA**.

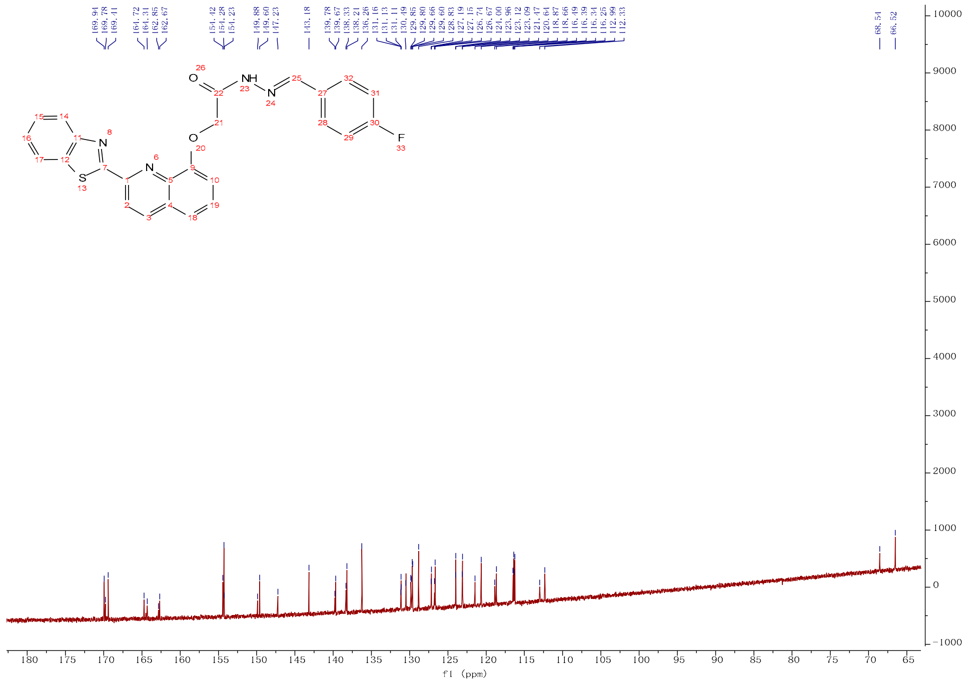

Figure. S13. ^{13}C NMR of compound **BQFA**.

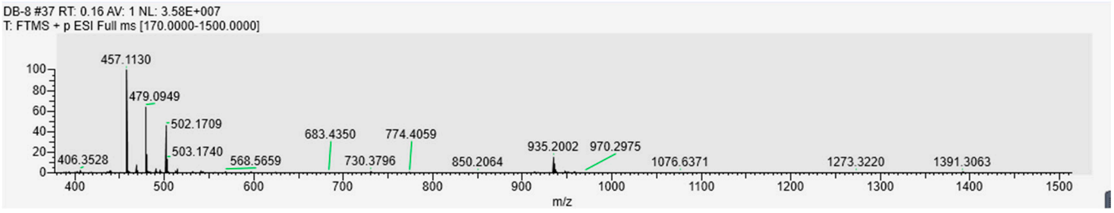

Figure. S14. HRMS of probe **BQFA**.

References

1. Praveen, P.A.; Saravanapriya, D.; Bhat, S.V.; Arulkannan, K.; Kanagasekaran, T. Comprehensive analysis of DFT-3C methods with B3LYP and experimental data to model optoelectronic properties of tetracene, *Materials Science in Semiconductor Processing.* **2024**,
2. Wang, K.; Wang, W.; Zhang, R.; Liu, Y.; Hou, C.; Guo, Y.; Zhang, C. Preparation of low molecular weight chondroitin sulfate from different sources by H_2O_2/ascorbic acid degradation and its degradation mechanism, *Food Chem.* **2024**, 434, 137392.
3. Liu, Y.T.; Zhang, Q.Q.; Yao, S.Y.; Cui, H.W.; Zou, Y.L.; Zhao, L.X. Dual-recognition "turn-off-on" fluorescent Biosensor triphenylamine-based continuous detection of copper ion and glyphosate applicated in environment and living system, *J Hazard Mater.* **2024**, 477, 135216.
